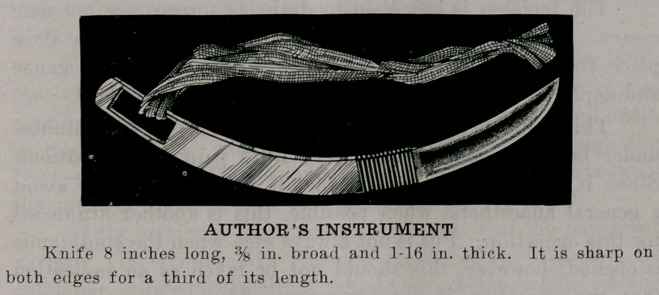# The Value of Epididymotomy

**Published:** 1914-09

**Authors:** E. P. Merritt

**Affiliations:** Urologist to the Atlanta Anti-Tuberculosis Association and Assistant in Urology at the Atlanta Medical College


### Journal-Record of Medicine

Successor to Atlanta Medical and Surgical Journal, Established 1855
and Southern Medical Record, Established 1870

**OWNED BY THE ATLANTA MEDICAL JOURNAL COMPANY**

PUBLISHED MONTHLY

*Official Organ Fulton County Medical Society, State Examining Board, Presbyterian Hospital, Atlanta, Birmingham and Atlantic Railroad Surgeons' Association, Chattahoochee Valley Medical and Surgical Association, Etc.*

EDGAR BALLENGER, M. D., Editor
BERNARD WOLFF, M. D., Supervising Editor
A. W. STIRLING, M. D., C. M., D. P. H.; J. S. HURT, B. Ph., M.D.
GEO. M. NILES, M. D., W. J. LOVE, M. D., (Ala.); Associate Editors
R. R. DALY, M. D., Associate Editor
E. W. ALLEN, Business Manager

**COLLABORATORS**

W. F. WESTMORELAND, M. D., General Surgery
F. W. McRAE, M. D., Abdominal Surgery
H. F. HARRIS, M. D., Pathology and Bacteriology
E. B. BLOCK, M. D., Diseases of the Nervous System
MICHAEL HOKE, M. D., Orthopedic Surgery
CYRUS W. STRICKLER, M. D., Legal Medicine and Medical Legislation
E. C. DAVIS, A. B., M. D., Obstetrics
E. G. JONES, A. B., M. D., Gynecology
R. T. DORSEY, Jr., B. S., M. D., Medicine
L. M. GAINES, A. B., M. D., Internal Medicine
GEO. C. MIZELL, M. D., Diseases of the Stomach and Intestines
L. B. CLARKE, M. D., Pediatrics
EDGAR PAULLIN, M. D., Opsonic Medicine
THEODORE TOEPEL, M. D., Mechano Therapy
A. W. STIRLING, M. D., Etc., Diseases of the Eye, Ear, Nose and Throat
BERNARD WOLFF, M. D., Diseases of the Skin
E. G. BALLENGER, M. D., Diseases of the Genito-Urinary Organs

VOL. LXI. ATLANTA, GA., SEPTEMBER, 1914. No. 6

### THE VALUE OF EPIDIDYMOTOMY.

BY E. P. MERRITT, M. D., UROLOGIST TO THE ATLANTA ANTI-TUBERCULOSIS ASSOCIATION AND ASSISTANT IN UROLOGY AT THE ATLANTA MEDICAL COLLEGE.

There are several different types of epididymitis, namely: gonorrheal, tubercular, syphilitic, etc. Of all the different types, gonorrheal will cover from 90 to 95% of cases. Therefore, I will discuss only this type tonight as it is the type with which we deal most often. There is no condition that will give a patient more pain than gonorrheal epididymitis. You may, without exaggeration, put it in the same class with renal colic so far as pain is concerned. This agonizing pain is relieved promptly by the surgical procedure, which I will mention later.

The mode of infection is the transmission of gonococci from the prostatic urethra into the ejaculatory ducts, thence to the seminal vesicles, and from there to the vas deferens, which leads to the tail of the epididymis. Usually the cause of this transmission is deep instrumentation, strong urethral injections, sexual intercourse, and other causes of unknown origin, while the patient has gonorrheal urethritis.

Gonorrheal epididymitis is usually unilateral and about 90% of the cases are confined to the epididymis alone, leaving the testicle intact, so far as the infection is concerned. However, the state of active hyperemia of the epididymis often causes the testicle to enlarge from pressure against it.

Text-books on Urology devote much space to the local treatment of gonorrheal epididymitis all of which has very little value after the disease has progressed to any extent. The chief operation advocated by them is that of Hagner, which is a good procedure, but rather extensive for several reasons:

1st. It cannot be done with any degree of satisfaction under local anaesthesia.

2nd. The testicle is unnecessarily exposed, and there are numerous other drawbacks to this operation.

The time to operate must, as in all other surgical procedures, be determined by the best judgment of the physician. Usually an operation is indicated as soon as the swelling is marked and the pain is severe. This occurs about 24 to 72 hours after there is pain in the testicle with radiation up the cord. An operation may be evaded in some cases if the patient is put to bed with the scrotum strapped up well and is given gonorrheal vaccine (mixed), urotropin, ice cap applied, together with the leaving off of all local injections. However, if the swelling and pain persist and increase an operation is imperative.

The course of gonorrheal epididymitis when treated in the usual way without resorting to surgical interference is from one to three weeks. With an epididymotomy the patient can usually get out of bed and attend to his duties in from two to five days. Note contrast. This procedure is believed by many to reduce the percentage of sterility. It seems that it would reduce this danger in almost every case from the following reasons: 1st. The part that is inflamed is opened and the inflammatory exudate has a chance to escape. 2nd. The blocking up of the tube is relieved when opened and drained. There are several other reasons which will not be discussed here. In practically all the cases the disease is unilateral and hence the question of sterility has not been worked out as thoroughly as would have been done otherwise.

**AUTHOR'S INSTRUMENT**

Knife 8 inches long, ⅜ in. broad and 1-16 in. thick. It is sharp on both edges for a third of its length.

The immediate relief that this operation brings to the patient is marvelous. I have for the past year in the clinic at the Atlanta Medical College done numbers of these operations with very gratifying results both to the patients and myself. The patients would come to the clinic with agonizing pain and after puncturing the epididymis which causes very little extra pain, would walk out thoroughly relieved. Dr. Champion and myself have done a number of these operations in the office under local anaesthesia with very gratifying results. The method I follow in doing this operation is very simple and satisfactory:

1. The patient is prepared by shaving the field for operation.

2. This is then painted with a 2 to 3% solution of iodine.

3. A 1% solution of cocain is injected in the skin for about ½ to 1 inch. Then push the needle straight into the tail of the epididymis so as to inject a few drops in it. This usually anaesthetizes the field for operation thoroughly.

4. The scrotum is firmly held with one hand, using care not to press too hard, on account of causing pain.

5. Make an incision with a sharp-pointed bistoury through the skin beneath the globus minor from ½ to 1 inch in length.

6. Then make several punctures in the globus minor with caution so as not to puncture the testicle.

The incision is left open to drain as sutures are not necessary. Next pack with a 5% iodoform gauze and leave it in place for twenty-four hours. Dress with plain sterile gauze and apply a "T" bandage so as to keep dressing in place.

This valuable operation may be done in a very few minutes under local anaesthesia with very little pain to the patient. Since it is universally agreed that it is always best to avoid a general anaesthetic when possible, this is another argument for this operation. Pus is not always seen when the epididymis is opened; however, this should not be taken as an indication that the operation is not necessary. There is usually more blood and bloody serum than pus. Occasionally pus is seen to flow freely in which gonococci may be demonstrated. In order to give patients the benefit of the very best method of treatment in cases of gonorrheal epididymitis, I would advise epididymotomy in all cases which have passed beyond the stage of immediate relief by other means.

## Figures and Tables

**Figure f1:**